# Complete Genome Sequencing of the Mouse Intestinal Isolate Escherichia coli Mt1B1

**DOI:** 10.1128/genomeA.00426-18

**Published:** 2018-05-24

**Authors:** Debora Garzetti, Claudia Eberl, Bärbel Stecher

**Affiliations:** a Max von Pettenkofer Institute of Hygiene and Medical Microbiology, Faculty of Medicine, LMU Munich, Munich, Germany; b German Center for Infection Research (DZIF), Partner Site Munich, Munich, Germany

## Abstract

Escherichia coli Mt1B1, a mouse isolate, is a facultative anaerobic bacterium which was shown to counteract Salmonella enterica serovar Typhimurium infection in a mouse model. In the present study, we describe the complete genome sequence of E. coli Mt1B1, composed of a 5.1-Mb chromosome and a 62.6-kb plasmid.

## GENOME ANNOUNCEMENT

Colonization resistance, defined as the ability of the gut microbiota to prevent infection by potential pathogens, is a key function fulfilled by the heterogeneous microbial community populating the mammalian gastrointestinal tract (1). To study this important feature, gnotobiotic mouse models can be employed for cause-effect analyses and to elucidate the role of individual bacteria (1). We have recently demonstrated that members of the Oligo-Mouse-Microbiota (Oligo-MM), a defined bacterial collection of 12 murine strains, confer partial colonization resistance against Salmonella enterica serovar Typhimurium (2). The addition of three facultative anaerobes, including Escherichia coli strain Mt1B1, to the Oligo-MM increased colonization resistance to the level of conventional mice. Importantly, Escherichia coli Mt1B1 showed high similarity to S. Typhimurium, both in terms of hierarchical KEGG module clustering and occupation of physical niches (2).

E. coli Mt1B1 (deposited at the German Culture Collection of Microorganisms and Cell Cultures [DSMZ] with number DSM-28618) has been isolated from the ileal mucosa of a conventional laboratory mouse, and its genome has been sequenced in frame of the mouse intestinal bacteria collection (miBC) (3). Analysis of the complete genome to discover specific metabolic functions and pathways is crucial for uncovering the interplay between E. coli Mt1B1 and the gut microbiota in the context of resistance against infection by enteric pathogens, like S. Typhimurium.

The complete genome of E. coli Mt1B1 was obtained by extracting the genomic DNA with a phenol-chloroform-based protocol from a culture grown overnight in LB medium, inoculated by a single bacterial colony. Sequences obtained on a PacBio RS II system with a 20-kb insert size (Eurofins Genomics, Ebersberg, Germany) were assembled using the Canu 1.7 software (4). The publicly available Illumina MiSeq reads (SRA accession number ERX1284231), after trimming and filtering with Trimmomatic 0.36 (5), were used for error correction by mapping them against the complete genome with BWA 7.10 (6), followed by extraction of the consensus sequence with CLC Genomics Workbench 7.5.1 (Qiagen Bioinformatics). The obtained genome sequence of E. coli Mt1B1 is composed of two contigs, with one chromosome of 5,115,630 bp and 50.6% G+C content, and one contig of 62,649 bp and 41.1% G+C content. The small sequence was found to be highly similar to plasmid pCROD2 (IncX4 type) from Citrobacter rodentium ICC168 and pCROD2-like plasmids from other E. coli mouse isolates by BLAST (7) and PlasmidFinder (8) searches. Gene prediction and subsystem analysis were performed using the Rapid Annotations using Subsystems Technology (RAST) server (9) for both the chromosome and plasmid sequences, resulting in 4,859 coding sequences, 22 rRNAs, and 86 tRNAs for the chromosome and 97 coding sequences for the plasmid. Genome-scale metabolic models were reconstructed with the model SEED (10), and the prediction of secondary metabolites and biosynthetic gene clusters was done using antiSMASH (11).

### Accession number(s).

The assembled complete genome sequence has been deposited in DDBJ/ENA/GenBank under the accession numbers CP028714 and CP028715.
